# Review of Multi-Modal Imaging in Urea Cycle Disorders: The Old, the New, the Borrowed, and the Blue

**DOI:** 10.3389/fneur.2021.632307

**Published:** 2021-04-28

**Authors:** Kuntal Sen, Afrouz A. Anderson, Matthew T. Whitehead, Andrea L. Gropman

**Affiliations:** ^1^Division of Neurogenetics and Neurodevelopmental Pediatrics, Department of Neurology, Children's National Hospital, George Washington University School of Medicine, Washington, DC, United States; ^2^Department of Research, Focus Foundation, Crofton, MD, United States; ^3^Department of Radiology, Children's National Hospital, George Washington University School of Medicine, Washington, DC, United States

**Keywords:** urea cycle disorders, neuroimaging, hyperammonemia, magnetic resonance spectroscopy, ornithine transcarbamylase deficiency, arginase deficiency, multimodal imaging, functional near infrared spectroscopy

## Abstract

The urea cycle disorders (UCD) are rare genetic disorder due to a deficiency of one of six enzymes or two transport proteins that act to remove waste nitrogen in form of ammonia from the body. In this review, we focus on neuroimaging studies in OTCD and Arginase deficiency, two of the UCD we have extensively studied. Ornithine transcarbamylase deficiency (OTCD) is the most common of these, and X-linked. Hyperammonemia (HA) in OTCD is due to deficient protein handling. Cognitive impairments and neurobehavioral disorders have emerged as the major sequelae in Arginase deficiency and OTCD, especially in relation to executive function and working memory, impacting pre-frontal cortex (PFC). Clinical management focuses on neuroprotection from HA, as well as neurotoxicity from other known and yet unclassified metabolites. Prevention and mitigation of neurological injury is a major challenge and research focus. Given the impact of HA on neurocognitive function of UCD, neuroimaging modalities, especially multi-modality imaging platforms, can bring a wealth of information to understand the neurocognitive function and biomarkers. Such information can further improve clinical decision making, and result in better therapeutic interventions. *In vivo* investigations of the affected brain using multimodal neuroimaging combined with clinical and behavioral phenotyping hold promise. MR Spectroscopy has already proven as a tool to study biochemical aberrations such as elevated glutamine surrounding HA as well as to diagnose partial UCD. Functional Near Infrared Spectroscopy (fNIRS), which assesses local changes in cerebral hemodynamic levels of cortical regions, is emerging as a non-invasive technique and will serve as a surrogate to fMRI with better portability. Here we review two decades of our research using non-invasive imaging and how it has contributed to an understanding of the cognitive effects of this group of genetic conditions.

## Introduction

**Figure 1:**
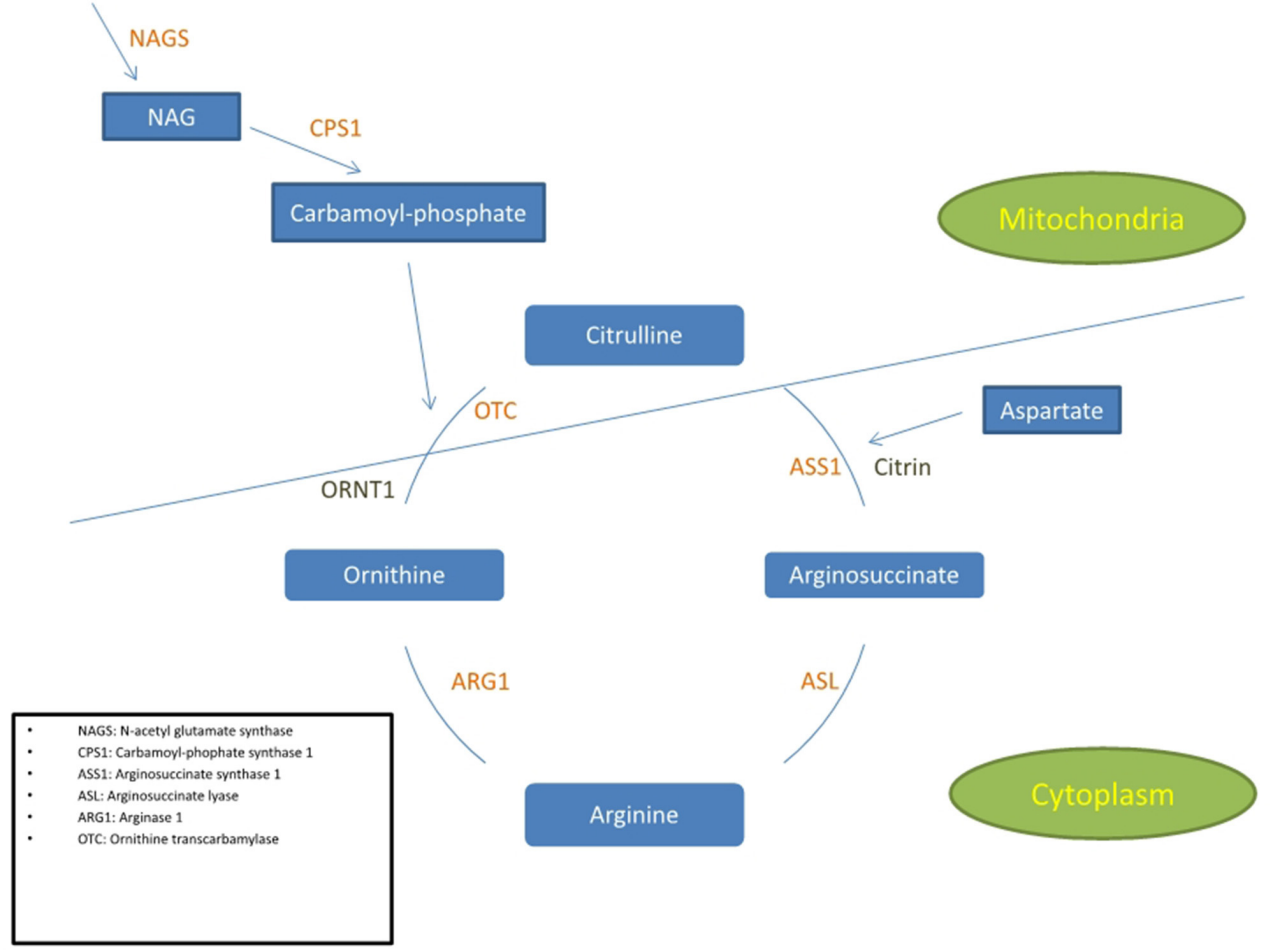
Simplified diagram of the urea cycle indicating the 6 enzymes and 2 transporters associated with UCD. The cycle is partly in the cytoplasm and partly in the mitochondria. The 3 enzymes positioned in the inner mitochondrial membrane are NAGS, CPS1, and OTC.

The urea cycle disorders (UCD) are rare disorders due to a deficiency of the enzymes or transport proteins that remove ammonia from the body (Figure 1). There are six enzyme deficiencies and two carrier defects that make up this group of conditions (1). The overall prevalence of the UCD is 1/35,000 which range from the more common Ornithine transcarbamylase deficiency (OTCD), an X-linked inborn error of metabolism (~1 in 56,500) to the least common being Arginase deficiency (~1:350,000 and 1:1,000,000). The remainder of the UCD, including arginase deficiency, are autosomal recessive. The UCD collectively are defined by a condition of protein intoxication (2). Arginase deficiency presents predominantly with a spastic paraplegia or quadriplegia. Late-onset UCD can present any time after the first 6 weeks of life and sometimes, for the first time in adulthood. Because many adult physicians are not familiar with the UCD, and because the clinical presentation may mimic other conditions that also present with acute confusional states or psychosis, they may be difficult to diagnose, if at all. The triggers of adult disease may be brought on by various metabolic stressors such as chronic starvation, gastric bypass surgery, rapid weight loss, and changes in diet with protein loading (3), or the postpartum period in addition to illness and certain medications (4–14). Since OTCD is X linked, female OTCD heterozygotes may show great variability in symptoms ranging from asymptomatic to significant clinical symptoms. It has been estimated that 5–20% of females ultimately develop symptoms however study of this group has shown that they manifest a similar albeit milder picture on magnetic resonance spectroscopy (MRS), diffusion tensor imaging (DTI), and cognitive testing (15).

While the brain may sustain the most impact from elevated ammonia, recent clinical observations has shown that the liver and kidney may be impacted in UCDs (16). Early and ongoing work in hepatic encephalopathy (HE) has laid the groundwork for knowledge of the impact of ammonia in the brain (17). Whereas, in HE, where the urea cycle enzymes are indiscriminately impacted due to liver cirrhosis which destroys the urea cycle enzymes by destruction of liver tissues, UCD are caused by selective enzyme deficiency.

Prior to 2004, the survival rate of patients with a UCD was 10% (unpublished data, Urea Cycle Rare Disease Consortium natural history study). Those who survived had significant neurological morbidity (18). Follow up neuroimaging studies in survivors of neonatal coma shows hypomyelination, leukodystrophy, myelination delay, and cystic changes as well as gliosis of the deep gray matter nuclei (19–25). Although UCD lead to significant encephalopathy, it was rare prior to 2005, that a patient had neuroimaging to understand the impact of HA on neurological function. After a government mandate, the rare disease clinical research network (RDCRN) was formed and the urea cycle rare disorders consortium (UCDC) was born. This allowed a deep dive into the function of UCD patients in a longitudinal manner and allowed a critical evaluation of therapies and neurocognitive outcomes. Currently with the development of newer therapies and improved clinical care, patients are living into adulthood. However, as patients live longer, they are noted to manifest varying degrees of neurocognitive difficulties particularly in the areas of working memory and attention, elements of executive function (26–30). We now understand that the downstream effects of HA and other toxic metabolites but quantification of these effects on cognitive performance during daily life activities is still unknown. How to prevent or mitigate these outcomes is a major challenge. No correlation exists between age of onset, genotype, peak ammonia level, neuroimaging, and/or phenotype. However, generally, degree of HA and its duration and the age of onset are predictive of outcome (31–35). Normal Intelligent Quotient (IQ) can occur after a hyperammonemic event although accumulating damage results in impairments that are irreversible (34–37).

The mechanism of ammonia induced neuropathology in UCD is not known. Accumulations of both ammonia and glutamate lead to toxic effects upon the brain. Glutamate is the main excitatory neurotransmitter and its sustained accumulation may be a causative mechanism of neuronal degeneration in a variety of neurological disorders (38). In animal models, the HA leads to excitotoxic cell death that eventually leads to the loss of NMDA receptors (39). In the OTCD sparse fur (Spf) mouse model, NMDA receptor is disrupted (40). The effects of Hyperammonemia and glutamate are astrocyte swelling (41, 42), seizures (28), increased permeability of the blood brain barrier, disrupted energy due to depletion of intermediaries of metabolism (29, 43), and altered levels of neurotransmitter and amino acid (44, 45).

### Basic Concepts in Multi-Modal Imaging

As neuroimaging technologies have become more sophisticated and accessible, the UCDC and others sought to use this non-invasive technology to understand the implications of HA on the brain. There is no one single MRI modality that allows a total appreciation of the entire picture of neurological damage OTCD. Therefore, multimodal brain imaging studies create a unique opportunity by providing a wealth of information and comprehensive framework to understand the brain and its injury pattern with each studied disorder. Longitudinal or cross-sectional imaging data have been reported in several adult onset degenerative conditions such as Parkinson disease, Alzheimer disease, and multiple sclerosis (46–48).

The traditional analysis in imaging has been to use Independent component analysis (ICA) (49) which serves as a general-purpose statistical model. One problem is that the estimated independent components might not be reliable and does not consider weighting of the components that may be more important. The goal is to extract the full complementary information each modality can provide to an understanding of the pathology (50). Combined Neuroimaging data from multiple imaging modalities can overcome the incomplete information from only individual modalities (50). Combination of methods such as electroencephalogram (EEG) that measures the neural electrical activity with that of fMRI and fNIRS that measure the hemodynamic response can provide complementary information about neurocognitive function and has shown to be effective in increasing the accuracy in seizure detection and classification (51, 52). Correlation analysis is used to find the association between several measurements such as electrophysiological activities using EEG and structural connectivity using DTI (53). Joint independent component analysis (jICA) is another multivariate method that allows combination of features from different modalities such as MRI and PET or fMRI and structural MRI to investigate the covariation across several datasets (54, 55). Classification techniques such as support vector machine (SVM) have also been used in biomarker identification of major depressive disorders using structural MRI measurement and diffusion MRI (dMRI) (56).

Merging information from different imaging modalities requires specialized post-processing tools that can merge data from the various modalities. One must consider that the image space of all meaningful measured qualities will likely vary for each imaging modality. One must determine how to combine the complementary features of each modality and what these are. This could be defined in terms of the underlying pathophysiology or in terms of a spatial and/or temporal resolution. There should be an added benefit in doing this vs. compared to analyzing and interpreting each dataset separately and independently from one another. An example of how this may work may involve the collection of diffusion tensor imaging (DTI), functional magnetic resonance imaging (fMRI), magnetic resonance spectroscopy (MRS), and functional near infrared spectroscopy (fNIRS) in ornithine transcarbamlyase deficiency (OTCD), one of the UCDs. Each of these modalities measures a different potential biomarker of an aspect of the disease fMRI and fNIRS measure the dynamics of the hemodynamic response based on neuronal activity during a task (57). Compared to fNIRS, fMRI can measure the hemodynamic response at depth of the brain, whereas fNIRS can measure the response at the cortical regions. The advantage of fNIRS is being portable and wearable as well as being less prone to motion artifact compared to fMRI. DTI can measure white matter integrity and structural connectivity (58). MRS can probe important information about metabolites that may be altered in relation to the disease (59).

## Current Imaging Modalities in UCD

### Structural Imaging and T1/T2/FLAIR

Magnetic resonance imaging has played the major rule in neuroimaging studies. This technique uses the magnetic field along with radio frequency waves to generate the image of the brain. The setup for MRI based technique include the tube-shape magnets, where patients lie inside the tube. Each MRI modality contributes to an understanding of some aspect of the pathology in UCD. MRI in late onset UCD patients shows diffuse cerebral edema and white matter lesions (60). Other MRI findings may include signal intensity increase in the regions of frontal lobes, cingulate gyri, temporal lobes, as well as the insular regions (60). Many patients may have a normal MRI with changes that appear after an acute hyperammonemic episode (61).

**Figure 2:**
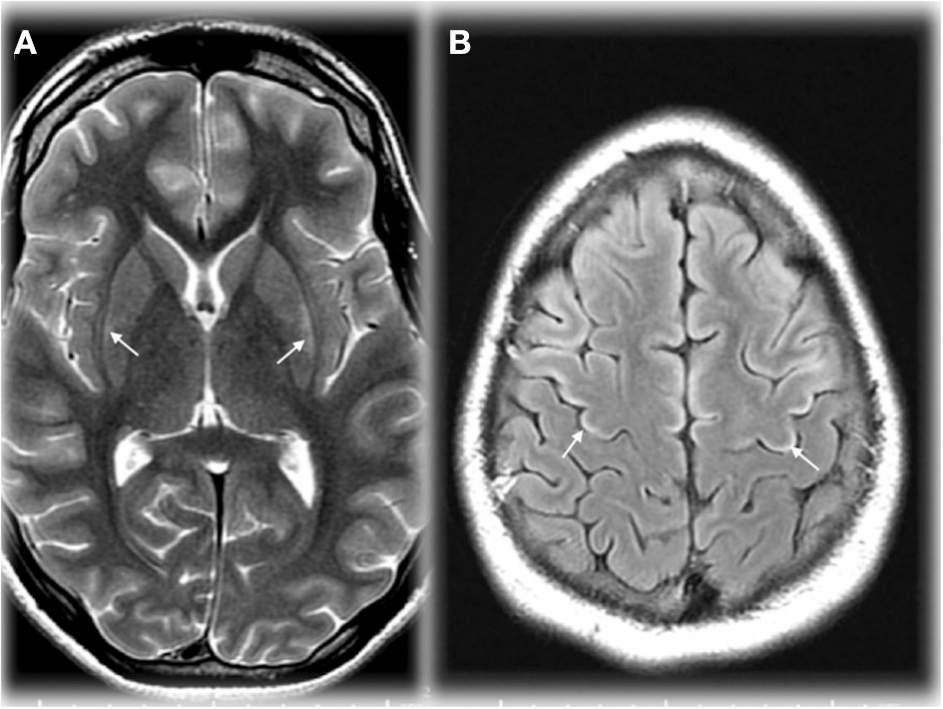
Axial T2 weighted image (TR/TE ms, 4,228/98) through the basal ganglia **(A)** demonstrating maximal changes in signal in the lateral putamen in an adolescent in OTCD after HA. Axial T2 FLAIR image (IT/TR/TE ms, 2,250/10,000/144) at the cerebral vertex **(B)** showing cortical hyperintensity in parts of the frontal lobes (motor strip, arrows). Reprinted from, with permission from IOS Press. Sen et al. (62).

Early in the course of HA, the MRI may be normal, and the only abnormal finding may be an elevated glutamine peak (gln) on MRS. MRS can detect elevated brain gln levels that is helpful in detection of subtle variations in OTCD females (59). Early diagnosis is critical to instituting ammonia lowering therapies, thus where available, 1HMRS adds typically 5–10 extra min to conventional MRI and can help in expediting diagnosis for partial or atypical OTCD when biochemical and molecular confirmation can take up to several days and weeks, respectively, and provides complementary information that can lead to early diagnosis (61) (Figure 2). At our institution, modern neuroimaging techniques play a role in clinical monitoring and clinical decision making.

### Diffusion Tensor Imaging (DTI)

DTI provides information regarding white matter tract such as its axonal density, myelination, and fiber orientation based on rate of diffusion of water molecules. Such information can be used to investigate the brain pathology in much greater detail than MRI. Given the normal early findings in patients with HA and those at baseline, we demonstrated that DTI is more sensitive in detecting white matter abnormalities in patients with OTCD that were normal-appearing using T2-weighted imaging (58). The extent of white matter abnormality also correlated with cognitive deficits, as we demonstrated key areas of difference underlie pathways important in executive function. The location of the frontal white matter deficits correlates with networks related to attention, executive function, and working memory that show deficiencies in this patient population (63). Specific details of imaging deserve mention. MRI may be normal by T1 and T2 early in the course of disease or with a short duration of HA. A predilection for diffusion signal changes in the insular and peri insular regions, occipital lobe, temporal lobe, basal ganglia, parietal lobe, and dorsal thalamus have been reported (64). DTI has been useful and informative in arginase deficiency (see section Neonatal Onset Urea Cycle Disorders).

### Magnetic Resonance Spectroscopy: 1H MRS

^1^HMRS produces metabolic spectra in a volume of cerebral tissue (voxel). This technique is based on analyzing the MR signal intensity at various frequencies induced by tissue chemical environment. MRS can therefore be used for tissue characterization such as studying the concentration of metabolites. A characteristic MRS HE profile as described by Cordoba et al. (65) showed an increase in the glutamine/glutamate level while indicating a decreased level of choline and myoinositol, in compensation for glutamine osmotic power.

### Magnetic Resonance Spectroscopy: 13C MRS

*In vivo* ^13^C spectroscopy is a method of MRS that can allow study of metabolic fluxes in brain (65–67). This requires infusing highly enriched 13-carbon substrates (acetate or glucose) along with complete proton decoupling that results in carbon signal enhancement by removing proton splitting. We explored the feasibility and safety of using ^13^C MRS on the frontal lobe of patients with OTCD as a proof of concept to evaluate low power direct detection of ^13^C enriched glutamate, glutamine, and bicarbonate to explore disorders of glutamine and glutamate neurotransmission and oxidative metabolism of frontal brain based disorders (68). Our group, furthermore, went on to use this method to determine turnover rate of cerebral glutamate in OTCD (69).

### Functional Near Infrared Spectroscopy (fNIRS)

In several recent reports, fNIRS has been used as a surrogate to fMRI imaging. fNIRS uses light in near infrared (NIR) range (700–900 nm) that is sensitive to changes of oxy-hemoglobin and deoxy-hemoglobin to probes the cortical regions. Typical fNIRS setup uses the pairs of sources and detector to send and detect the changes in the light intensity, respectively. Since the blood absorbs the light in the NIR region, the detected changes in the light intensity are correlated with the cortical hemodynamic due to neurovascular coupling. Therefore, the fNIRS measurements are like that of the fMRI Blood Oxygenation Level Dependent (BOLD) signal. The system is wearable, where the array of sources and detectors in the shape of cap or headband are worn on the head. Advantages of this methodology are its portability and the fact that it avoids the claustrophobia of the MRI scanner which limits imaging young, cognitively impaired and claustrophobic patients and imaging can be done in realistic setting (70, 71). Another apparent benefit in this group is the fact that it is not degraded by movement. fNIRS therefore can be used to advantage in research on infants or more cognitively impaired individuals (71, 72).

**Figure 3:**
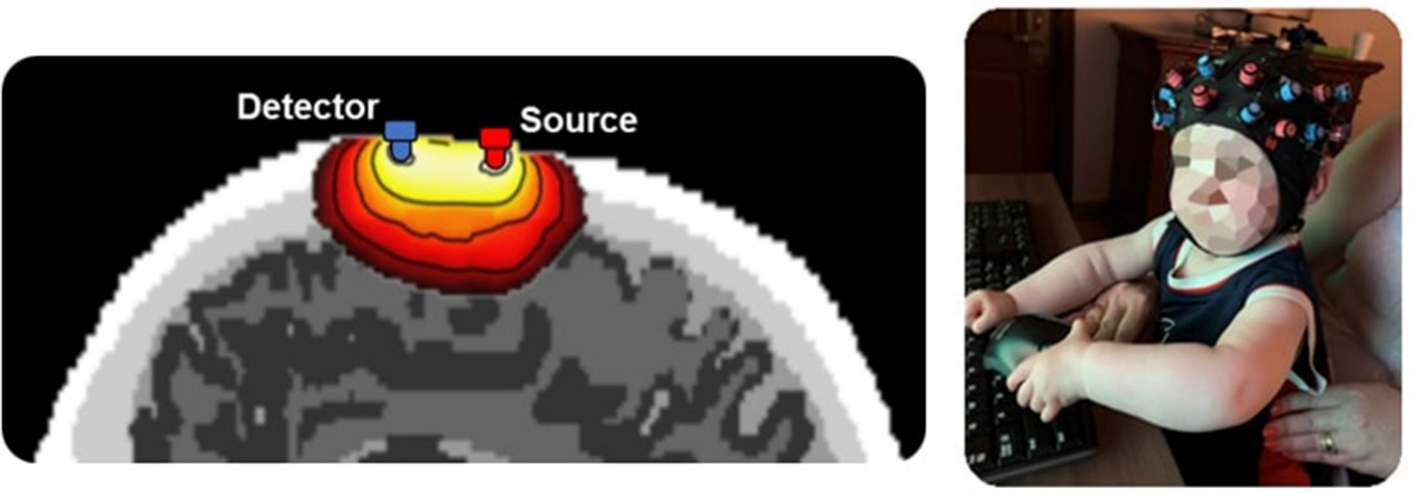
Left – A diagram of near infrared light probing from location of source through cortical region. Light intensity that is backscattered is measured by a detector. Changes in the light intensity as measured by the detector will be converted to changes in oxy-hemoglobin and deoxy-hemoglobin. Right – Several combinations of sources and detectors are used to cover the cortical regions.

fNIRS assesses local changes in cerebral hemodynamic levels of cortical regions associated with brain activity. Compared fMRI and positron emission tomography (PET), fNIRS has better temporal resolution (72, 73) and unlike PET it is non-invasive (Figure 3).

## Neuroimaging Clinical Studies in UCD

### Arginase Deficiency

As a distal UCD defect, arginase deficiency most commonly presents in childhood or adulthood with spasticity, cognitive impairment, and/or seizures, and less commonly, acute encephalopathy (63, 74, 75). Structural brain abnormalities are non-specific and include variable atrophy, deep and/or subcortical cerebral white matter hyperintense lesions on T2WI, and reduced fractional anisotropy in the corticospinal tracts (63, 74–80). Mild striatal lesions may be present (63, 77). These changes may reflect arginase induced, NOS-mediated oxidative damage (63). Furthermore, putaminal T1 shortening can occur (77). In the later onset forms, MRS may be normal or show mild changes including arginase elevation at 3.8 ppm, elevated glutamine, and decreased myoinositol. Rarely, arginase deficiency (63, 80) can present earlier with hyperammonemic induced encephalopathy; in those cases, MRI findings are similar to those of proximal UCD including cerebral swelling/edema which may spare the thalami (80). Reduced diffusion may occur in the sub-insular and other subcortical white matter and capsules during active injury (80). Severe injury can even lead to multifocal cystic encephalomalacia (81). MRS in early onset arginase deficiency shows changes like those of early onset proximal UCD. Namely, elevated glutamine and glutamate and decreased myoinositol secondary to hyperammonemia (80).

**Figure 4:**
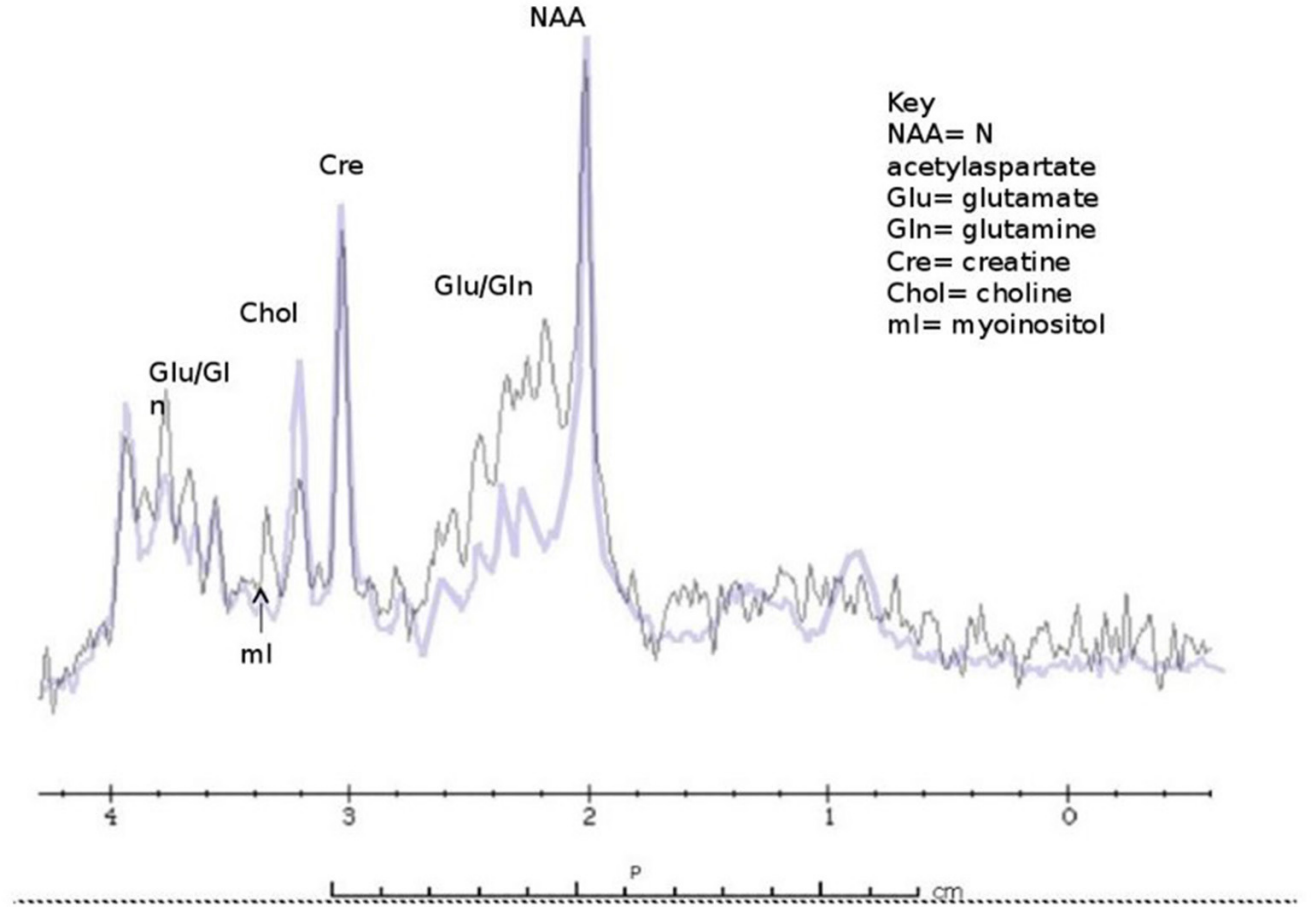
Classic findings of 1H MRS in OTCD - elevated glutamine peak with reduced myoinositol and choline and peaks (purple) compared to normal subject (black).

We documented marked depletion of myoinositol in asymptomatic and symptomatic subjects with partial OTCD relative to age matched controls as well as elevations of gln (59) (Figure 4). We therefore believe that these parameters will prove to be solid indicators of the effect of HA on brain metabolism and will predict the response to therapy. The classic triad in proximal UCD (Carbamoyl Phosphate Synthetase I deficiency and OTCD) consists of elevated glutamine peak with reduced myoinositol and choline peaks. In Arginase deficiency, glutamine peak is elevated followed by normal myoinositol and normal or increased choline. Based on our unpublished data, increased guanidino peaks are observed in Arginosuccinate Synthetase and Arginosuccinate Lyase deficiency (unpublished data, Urea Cycle Rare Disease Consortium).

### Neonatal Onset Urea Cycle Disorders

**Figure 5:**
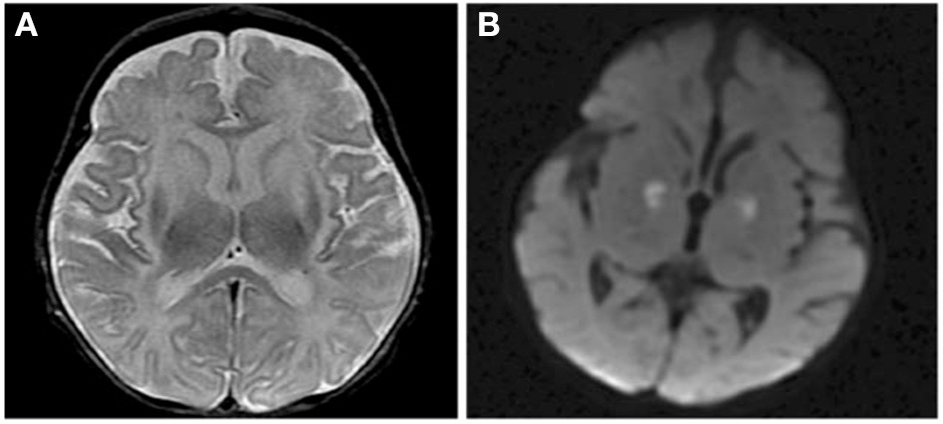
Axial T2 **(A)** and diffusion brain MR **(B)** images through the basal ganglia from a 13 day old female with CPSD1 deficiency. Symmetric basal ganglia and insular cortex hyperintensity reflects edema and laminar necrosis in the setting of subacute hyperammonemic injury. Reduced diffusion in the globi pallidi may represent more acute injury due to secondary energy failure or subacute pre-wallerian degeneration.

Neonatal brain MRI interpretation is predicated on knowledge of the patient's corrected gestational age, symptoms (type, onset, and duration), and whether other mimics such as ischemia and infection have been excluded clinically. Neonatal onset UCD related hyperammonemia causes variable brain edema/injury that tends to correlate with severity and duration, but not with the underlying defect. As the clinical severity increases, lesional location progresses from regional to diffuse in the following manner: periinsular, frontal, parietal, temporal, then occipital lobes (62). This results in two main neuroimaging patterns of neonatal onset UCD: (1) diffuse and (2) central. The more severe diffuse pattern involves the cerebral cortex and basal ganglia extensively, and occasionally, the thalami and brainstem rendering it indistinguishable from hypoxic-ischemic encephalopathy (HIE) and encephalitis (62). Typically, infants with diffuse cerebral involvement had higher plasma glutamine levels and worse neurological outcomes (62). The central pattern is more specific to UCD based on selective vulnerability of brain tissue. This signature appearance includes symmetric edema (vasogenic, cytotoxic, and intramyelinic) in the insular/periinsular, sylvian/perisylvian, perirolandic, and basal ganglia regions (62). Importantly, the thalami are typically spared which helps exclude HIE. Diffusion abnormality extent (diffuse cortical and/or striatal) trends negatively with prognosis (82) (Figure 5). However, not much is known about the expected duration of diffusion abnormalities in and after a hyperammonemic event which may be mechanistically different from those of hypoxic ischemic injury. Laminar necrosis rapidly develops with hyperintense signal on T1 weighted image (T1WI) in the involved cortex in subacute injury. Chronic injury shows encephalomalacia, gliosis, myelination disturbance, and sometimes, cystic necrosis. During metabolic decompensation, MRS shows increased glutamine and glutamate, decreased myoinositiol, and in severe cases, lactate.

### Pre-frontal Cortex Activation in OTCD

**Figure 6:**
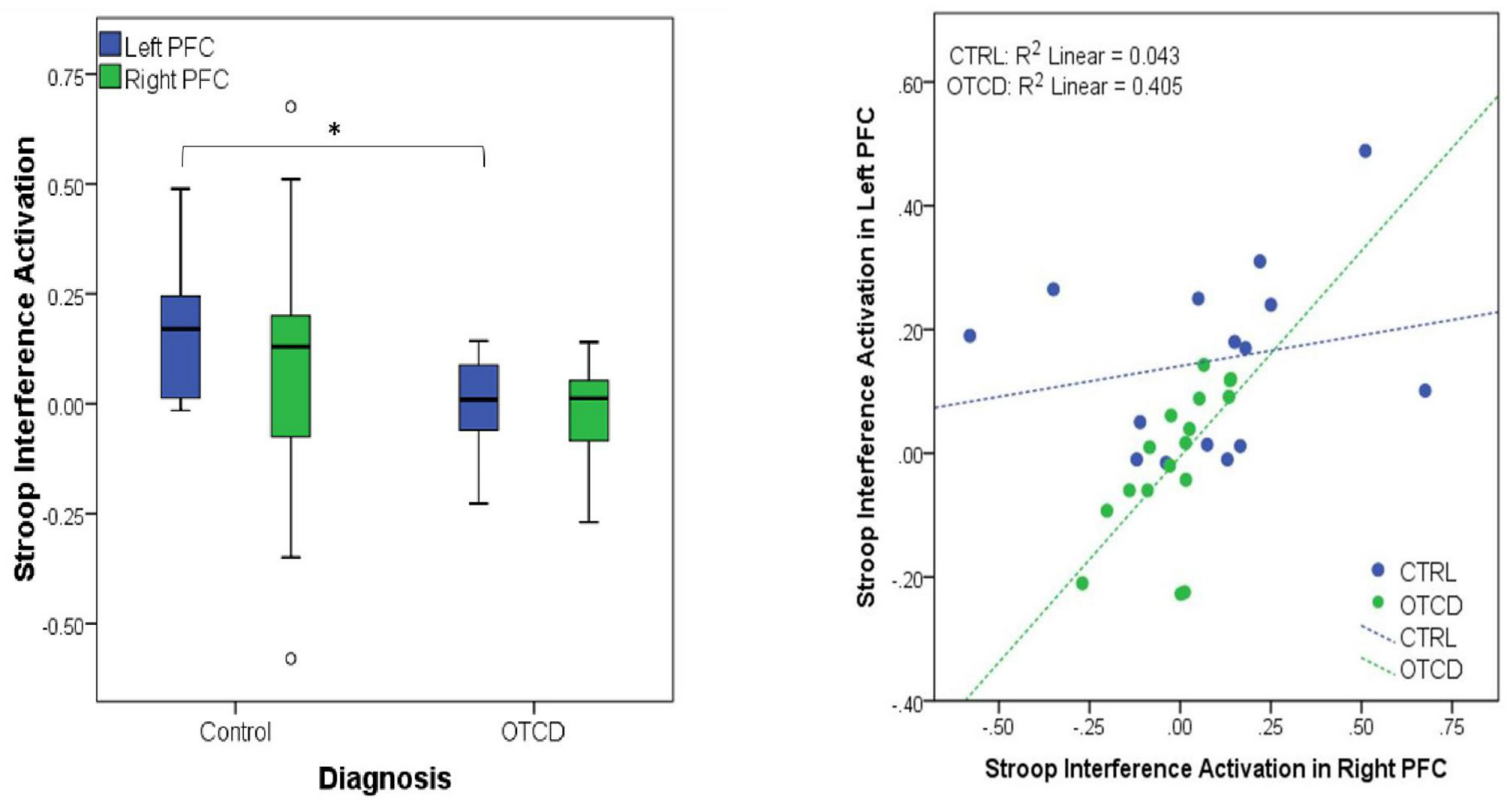
(Left) Significant activation difference between controls and participants with OTCD in Left PFC. (Right) Significant correlation between left and right PFC in OTCD participants (https://www.sciencedirect.com/science/article/abs/pii/S1096719219307310).

fNIRS is ideal to evaluate brain activity in inborne error of metabolism at baseline and during recovery of an acute metabolic event. Our prior fMRI experience in OTCD demonstrated altered brain networks in areas underlying executive function in frontal lobbe (83). We expected fNIRS to provide a sensitive measure of cortical hemodynamics. Using fNIRS, we measured the cortical activation of the pre-frontal cortex (PFC) while participants performed a Stroop task. We showed that in control participants left PFC had higher task related activation compared to patients with OTCD (*F*_(1, 27)_ = 6.15, *p* = 0.02). In subjects with OTCD, we further demonstrated a significant correlation between cortical activation in left and right pre-frontal cortex (PFC), where patients with OTCD showed bilateral increase in PFC activation (*r* = 0.62, *p* = 0.01) but such trend was not observed in control subjects (*r* = 0.23, *p* = 0.42) (57) (Figure 6).

## Imaging Recovery of HA

**Figure 7:**
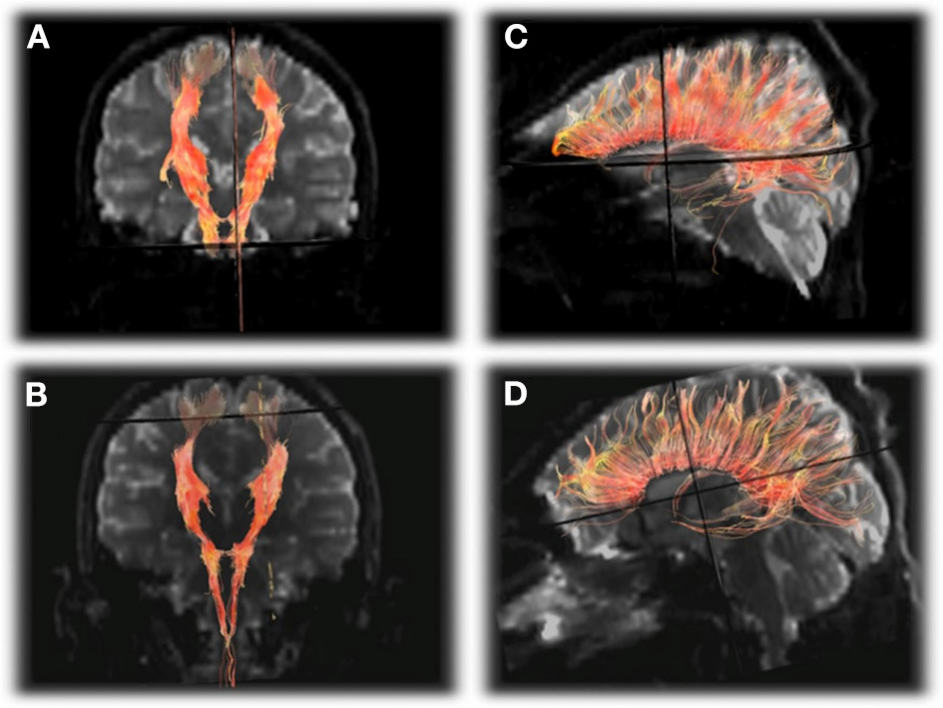
DTI tractography of the corticospinal tracts displayed in the anterioroposterior plane **(A,B)** and corpus callosum displayed laterally **(C,D)** from the first exam **(A,C)** and final exam **(B,D)** showing subtle decrease in the number of projectional and commissural fibers over time. Reprinted from, with permission from IOS Press. Sen et al. (62).

Although there are several reports of multimodal MRI at baseline or during acute HA, less is known about the recovery of HA with regard to changes in time and space. A prospective multimodal sequence of an OTCD patient with MRI/MRS exams performed during (5 over 1 month) and after (3 and 5 months) a hyperammonemic episode demonstrated abnormal signal changes in the posterolateral putamen, and supplemental motor strip (SMA), pre-motor cortex, and post-central gyrus (61). This occurred with cerebral hypoperfusion evolving to hyperperfusion. Apparent diffusion coefficient values increased while fractional anisotropy decreased in key areas such as cingulum corpus, callosum, and projectional fibers which correlated with reduction in volume of white matter fibers on tractography (Figure 7). Elevated glutamine and reduced myoinositol were present during HA on MRS. Chronic imaging changes reflecting permanent injury were found long after the apparent clinical resolution despite normal ammonia and plasma glutamine levels (61). This is not surprising since many patients report residual neurocognitive slowing following HA recovery. This study demonstrated the long-term effects and altered structural and metabolic sequence despite resolution of plasma markers of altered metabolism (61).

## Conclusions

The goal of UCD therapy is to decrease morbidity of the brain, understanding its impact on injury pattern, time course and evolution to frame treatment and long-term monitoring in patients. Injuries to the native pathways, and/or development of an additional accessory pathway can arise differential neural networks. More quantitative features such as axonal integrity and microscopic features of myelination are best evaluated using DTI and DWI, instead of T1 and T2 weighted changes in white matter that can detect a macroscopic amount of damage.

This manuscript reviews two decades of our work encompassing multi modal neuroimaging in UCD and provides a glimpse into the future techniques which will help to further delineate our understanding of this group of disorders. Information ranging from alterations in structure (T1/T2/FLAIR) to fluctuations in metabolites (^1^H and ^13^CMRS) have led to unraveling of disease pathophysiology, helped in expediting diagnosis as well as monitoring effects of therapeutic interventions. Imaging attributes have helped in expanding the clinical spectrum and distinguishing between partial and complete enzyme deficiencies, OTCD hemizygotes and heterozygotes as well as proximal and distal UCD. Modalities which elucidate dynamic neural activity such as EEG, fMRI, and fNIRs, when combined with tractography and perfusion studies can provide unprecedented insights and broaden the cognitive phenotypes associated with these neurometabolic disorders. Multimodality studies, such as combined fNIRS, EEG, and fMRI can provide information about hemodynamic and electrical neural activity to further clarify the underlying neurocognitive function and its impairments in UCD. Addition of machine learning methods to these modalities can facilitate clinical decision making and finding the neural signatures of UCD. Studying these temporal events in the central nervous system of patients with UCD has identified the different signature patterns of HA and can be used as a model for neuroimaging research in other inborn errors of metabolism.

## Author Contributions

KS and AA wrote sections of the manuscript and edited the manuscript. MW wrote sections of the manuscript, supplied figures, and edited the manuscript. AG conceptualized the manuscript, acquired data that is referenced in the manuscript, wrote the first draft, and edited all subsequent drafts. All authors contributed to the article and approved the submitted version.

## Conflict of Interest

The authors declare that the research was conducted in the absence of any commercial or financial relationships that could be construed as a potential conflict of interest.

## Abbreviations

DTI: Diffusion tensor imaging
fMRI: functional Magnetic resonance imaging
fNIRS: Functional Near Infrared Spectroscopy
gln: glutamine peak
HE: hepatic encephalopathy
HA: hyperammonemia
HIE: hypoxic-ischemic encephalopathy
IEMs: inborn errors of metabolism
ICA: Independent component analysis
MRI: magnetic resonance imaging
MRS: magnetic resonance spectroscopy
NBS: newborn screening
OTCD: ornithine transcarbamylase deficiency
PET: positron emission tomography
PFC: pre-frontal cortex
RDCRN: rare disease clinical research network
SMA: supplemental motor strip
UCD: urea cycle disorder
UCDC: urea cycle rare disorders consortium.
